# Effects of *Dendrobium* Polysaccharides on the Functions of Human Skin Fibroblasts and Expression of Matrix Metalloproteinase-2 under High-Glucose Conditions

**DOI:** 10.1155/2021/1092975

**Published:** 2021-03-08

**Authors:** Yajia Li, Ziqin Cao, Qiangxiang Li, Chenxu Wang, Zhuo Zhou

**Affiliations:** ^1^Department of Dermatology, Xiangya Hospital, Central South University, Changsha, China; ^2^Department of Orthopaedics, The Second Xiangya Hospital, Central South University, Changsha, Hunan, China; ^3^National Clinical Research Center for Geriatric Disorders of Xiangya Hospital, Central South University (Sub-Center of Ningxia), Yinchuan, Ningxia Hui Autonomous Region 750001, China; ^4^Ningxia Geriatric Disease Clinical Research Center, People's Hospital of Ningxia Hui Autonomous Region, Yinchuan, Ningxia Hui Autonomous Region 750001, China; ^5^Hunan People's Hospital, Department of Hunan Institute of Geriatrics, Changsha 410002, China

## Abstract

The effects of *Dendrobium* polysaccharides (PDC) on the functions of human skin fibroblasts (HSFs) and expression of matrix metalloproteinase-2 under high-glucose conditions and exploration of the underlying mechanism remain unclear. We used the 3-(4,5-dimethylthiazol-2-yl)-2,5-diphenyltetrazolium bromide (MTT) analysis and flow cytometry to evaluate the cell viability and apoptosis. The collagen levels were determined by the Sircol™ Collagen Assay. Real-time quantitative polymerase chain reaction (RT-PCR) was used to detect the expression of matrix metalloproteinase-2 (MMP-2) and matrix metalloproteinase inhibitor (TIMP-2) mRNA. We found the following: (1) under the high-glucose condition, the HSF cell viability, the expression of TIMP-2 mRNA, and the collagen levels were reduced, while the apoptosis rate and the expression of MMP-2 mRNA increased (*P* < 0.05). (2) In the high-glucose + PDC group, the PDC reversed the changes in the collagen level, viability, and apoptosis rate of the HSF cells caused by high glucose, with the expression of protein and TIMP-2 mRNA increased and the level of MMP-2 mRNA decreased (*P* < 0.05). This is the first time attempting to reveal that PDC can exhibit protective effects on HSF under high-glucose conditions, which may be related to the upregulation of the TIMP-2 expression and inhibition of the MMP-2 expression.

## 1. Introduction

The skin of diabetic patients is known to be susceptible to damage and does not heal easily following an injury. Diabetic skin lesions are related to diabetic vascular disease, neuropathy, cell dysfunction, and abnormal cytokine secretion; however, the specific mechanism for their formation remains unknown [1, 2]. Thus, evaluation of the molecular mechanism leading to the development of diabetic skin ulcers and the determination of appropriate interventions are of great significance for the diagnosis and effective treatment of this condition. Human skin fibroblasts (HSFs) are a type of repair cells in the dermis and play an important role in moderate tissue metabolism. Cells of this type also secrete collagen as well as noncollagen components, such as the outer matrix, and play a critical role in wound healing [3, 4]. In addition, they are important in maintaining cell elasticity, cultivating water, and supporting epidermal cells [5–8]. It has been determined that fibroblasts are crucial effector cells in the processes related to diabetic skin lesions and participate in the entire wound repair process. The changes in the biological characteristics of these cells are fundamental to the development of diabetic skin lesions. It is noteworthy that the decrease in the number and activity of fibroblasts is also one of the reasons for the reduced synthesis of collagen [9, 10]. Many in-depth studies have shown that the amount and vitality of skin fibroblasts are also important in diabetic skin injury and healing [11, 12]. *Dendrobium* polysaccharide, also called polysaccharides of *Dendrobium candidum* (PDC), is the main bioactive substance of *Dendrobium candidum*. Previous studies confirmed that PDC could inhibit islet cell apoptosis and necrosis, protect islet cells, and prevent diabetes. In addition, they have also been shown to prevent calcium overload and inhibit corneal epithelial cell apoptosis. PDC prevent skin photoaging; therefore, they display protection and repair properties [13–15]. However, the effects on human skin fibroblasts under high-glucose conditions are yet to be confirmed. Consequently, in the present study, we used various concentrations of PDC to treat HSF *in vitro* under high-glucose (HG) conditions. We observed the effects of the PDC on the activity and apoptosis of HSF, as well as on the expression of matrix metalloproteinase-2 (MMP-2). The key aim was to establish the protective properties of the PDC on HSF in diabetes and to provide new directions for the treatment of diabetic skin lesions.

## 2. Materials and Methods

### 2.1. Experimental Materials

Trypsin, Dulbecco's Modified Eagle's Medium (DMEM) culture solution, and low-glucose DMEM culture medium were obtained from Gibco (USA). Fetal bovine serum was purchased from Gibco (USA) or Hangzhou Sijiqing Biological Engineering Material Co. Ltd. (China). Medium-strength protein lysate, NA-Red, and Ponceau S solution were purchased from Biyuntian Biotechnology (China). Biocolor Sircol Soluble Collagen Assay was purchased from Biocolor (UK). The polysaccharide of *Dendrobium candidum* (PDC) was prepared in house. The CO_2_ incubator was obtained from Wuhan Huahai or Olympus (China). The KH-600DB data ultrasonic cleaner was purchased from JBtek (USA), while the BD FACSCalibur flow cytometer was obtained from Becton Dickinson (USA).

### 2.2. Extraction of Polysaccharide of *Dendrobium candidum*

The extraction process of polysaccharides from the protocorm of *Dendrobium officinale* has been studied. The process in our study is as follows: raw material, weighing, hot water extraction, rough filtration, evaporation concentration, filtrate standing, vacuum filtration, water bath concentration, freeze-drying, and crude polysaccharide. 2000 g fresh raw materials of *Dendrobium officinale* were weighed and cut into small sections to facilitate the extraction of polysaccharides. 200 g of them were reserved for freeze-drying. The remaining parts were extracted by hot water several times. According to the ratio of medicine and water, the medicine was extracted with hot water (medicine : hot water = 1 : 4) for 2 hours, and then the initial extraction solution is reserved, then the mixture was extracted (initial extraction solution :water = 1 : 2) for next one hour. Then, the filtrate was evaporated and condensed to a certain volume, and then it was freeze-dried for 24 hours to obtain the crude polysaccharide. The extraction technology of polysaccharides from the protocorm of *Dendrobium officinale* was optimized. The extraction conditions were established by orthogonal design L (934). The experiment was repeated three times. The crude polysaccharide from the protocorm of *Dendrobium officinale* was extracted by optimized technology, and the purity was 51.3%. In the experiment, the crude polysaccharide of the *Dendrobium candidum* protocorm can be dissolved in three distilled water to prepare the initial solution of 10 mg/ml, which is filtered and sterilized. Before use, the medium is added and diluted to the required concentration.

### 2.3. Primary Culture of HSF

The procedure was conducted based on the previously reported method [16]. The infant foreskin was aseptically removed and treated with penicillin 100 *μ*g/mL and streptomycin 100 *μ*g/mL. It was then thoroughly washed with phosphate-buffered saline (PBS) and D-Hanks' solution. After removing the subcutaneous tissue, the skin specimen was cut into small pieces using sterile ophthalmic scissors and placed in a type II collagenase digestion solution at 4°C for overnight digestion. The epidermis and dermis were aseptically separated. The epidermis was discarded, whereas the dermis was cut and transferred to a solution containing 0.25% trypsin (excluding ethylenediaminetetraacetic acid (EDTA)) for approximately 15 min. The digestion was stopped by the addition of a high-glucose DMEM medium containing 10% calf serum. The solution was centrifuged at 800 g for 5 min, and the resulting pellet was inoculated into a 25 mL plastic culture flask. Approximately 4 mL of high-glucose DMEM culture solution containing 10% calf serum was carefully added as not to float the tissue. The culture flask was placed in a 37°C, 5% CO_2_ incubator overnight [16]. The following day, 5 mL of the medium was added. The primary cells were passaged when they were >80% full. During the passage, the cells were digested with 0.25% trypsin (excluding EDTA). The culture flask was patted during the retraction of the cell body under an inverted microscope before the addition of 10% calf serum. The digestion was stopped by the addition of the high-glucose DMEM medium. The cells on the wall of the flask were gently pipetted and collected into a 10 mL centrifuge tube. The cells were centrifuged at 800 g for 5 min, and the supernatant was discarded. The cells were inoculated and routinely cultured.

### 2.4. High-Glucose Model Establishment and Experimental Grouping

The HSF cells were cultured and passaged. When the cells reached 80% confluence, the serum-containing culture medium was discarded. The serum-free medium was changed after 24 h, and cultures containing different concentrations of glucose were subsequently prepared (5, 10, 15, 20, 25, 30, 35, and 40 mmol/L of glucose). After observation at 12, 24, 36, 48, and 72 h, the optimal glucose concentration for the high-glucose model of the HSF cells was established at 25 mmol/L. The experiment was divided into the following groups: (1) control group (control, C): HSF cells were cultured with the DMEM medium containing 10% fetal bovine serum for 48 h; (2) high-glucose group (high glucose, HG): HSF treated with the culture solution containing 25 mmol/L of glucose for 48 h; (3–5) PDC at different doses: HSF treated with the culture solution containing the 100, 200, and 400 *μ*g/mL of PDC for 48 h; and (6–8) high glucose + different doses of the PDC group (HG + PDC): HSF treated with the culture solution containing 25 mmol/L of glucose and 100, 200, and 400 *μ*g/mL of PDC for 48 h. High mannitol (25 mmol/L) was utilized as the osmotic pressure control.

### 2.5. MTT Cell Viability Test

The 3-(4,5-Dimethylthiazol-2-yl)-2,5-diphenyltetrazolium bromide (MTT) method was used to detect cell viability. The principle of this assay is that the dehydrogenase enzymes in living cells reduce tetrazolium to a water-insoluble blue product, i.e., formazan, which precipitates in the cells. Conversely, dead cells do not exhibit this property. Dimethyl alum dissolves the blue-purple crystals in the cells, and the color depth is proportional to the amount of formazan [17, 18].

### 2.6. Collagen Detection Method

The cell supernatants of each group were collected for the determination of collagen content in the cell culture medium. 100 *μ*L of the culture medium containing collagen was separated, and the concentrated reagents were added. The supernatant was discarded following centrifugation at 4°C overnight. Standard collagen was diluted to 0, 0.01, 0.05, 0.1, 0.2, and 1.00 mg/L, respectively. 500 *μ*L of Sircol dye was added to each sample and incubated for 30 min. The samples were then washed with Acid-Salt Wash Reagent (R). 250 *μ*L of alkaline metal reagent was added to each tube before mixing by the vortex. 200 *μ*L of the sample was transferred onto a 96-well plate, which was analyzed by detection utilizing a microplate reader. The detection wavelength was 555 nm, and the absorbance value was adjusted to zero with water. The absorbances of blank reagents, standard collagen, and test samples were measured three times for accuracy. The test error was ±10%.

### 2.7. Detection of the Effect of PDC on the Apoptosis of HSF in the HG Environment Using Annexin V-FITC/PI and Flow Cytometry

The cells of the nine groups were separated during the logarithmic growth period. Following digestion by pancreatin without EDTA, the cells were placed in centrifuge tubes and rinsed twice with PBS. The cells were subsequently centrifuged for 5 min at 800 g. The cells were collected and placed in 1 mL Eppendorf tubes. 500 mL of binding buffer was added to suspend the cells. 5 mL of annexin V-FITC and 5 mL of propidium iodide were added and allowed to react for 5–15 min at room temperature.

### 2.8. Determination of the Effect of PDC on the Expression of TIMP-2 mRNA and MMP-2 mRNA in the HSF Cells in the HG Environment Using RT-qPCR

Nine groups were treated according to the described procedures. The cells were collected to determine the expression of TIMP-2 mRNA and MMP-2 mRNA in the HSF cells using the real-time quantitative polymerase chain reaction (RT-qPCR). The extraction of the total RNA from the HSF cells, as well as the reverse transcription of RNA and RT-qPCR, was carried out utilizing the SYBR® method. The target gene sequence was searched in the National Center for Biotechnology Information (NCBI). Primer 5 software was used to design the primer. System composition and amplification of quantitative PCR were conducted according to the method previously described by Megha [19] (Table 1).

### 2.9. Statistical Treatment

All the data were analyzed using the SPSS version 19.0 software package. The measurement data were presented according to the differences between the average ± standard values. The *t*-test of the individual samples was performed to compare the average values between two groups satisfying the normal distribution and homogeneity of variance. Significance between groups was also evaluated by one-way analysis of variance (ANOVA) followed by a Tukey HSD post hoc test. The single-factor analysis of variance was used for the comparison of average values among the various groups. Findings were given as mean ± SD and compared by Dunnett's test. All groups were compared in pairs. *P*<0.05 indicated that the difference was of statistical significance.

## 3. Results

### 3.1. Effects of High Glucose Concentration and PDC on the Viability of the HSF Cells

**Figure 1:**
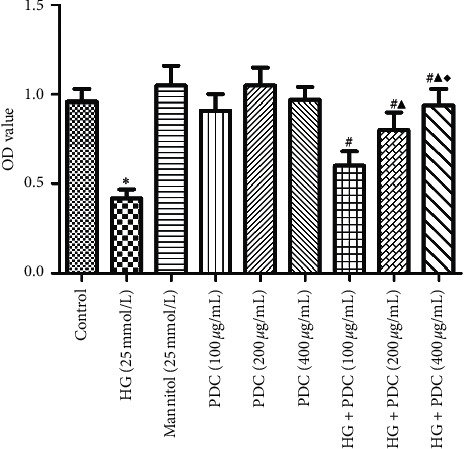
Effects of high glucose concentration and PDC on the viability of the HSF cells. HSF cells were incubated at high glucose concentration (25 mmol/L) or PDC (100, 200, and 400 *μ*g/mL) for 48 h. The MTT assay was used to detect the cell viability. All the data were expressed as $\bar{x}\pm S$ (*n* = 3). ^*∗*^*P* < 0.05, compared with the control group; ^#^*P* < 0.05, compared with the high-glucose group; ^▲^*P* < 0.05, compared with the high-glucose + *Dendrobium* polysaccharide (100 *μ*g/mL) group; ^♦^*P* < 0.05, compared with the high-glucose + *Dendrobium* polysaccharide (200 *μ*g/mL) group.

The obtained results are demonstrated in Figure 1 and Table S1. As can be seen, compared with the control group, the HSF cell viability of the high-glucose group was significantly reduced (*P*<0.05). On the contrary, the cell concentration of the analogous glucose content group was not significantly different from the control group (*P*>0.05), indicating that high glucose concentration leads to reduced viability of the HSF cells. Moreover, this outcome also suggests that induced cell damage is not related to the osmotic pressure. No significant difference in the cell viability between the *Dendrobium* polysaccharide (100, 200, and 400 *μ*g/mL) and the control groups was observed (*P*>0.05). Compared with the high-glucose group, the cell viability of the high-glucose + PDC (100, 200, and 400 *μ*g/mL) groups significantly increased, demonstrating a concentration dependence (*P*<0.05).

### 3.2. Effects of High Glucose Concentration and PDC on HSF Cell Apoptosis

**Figure 2:**
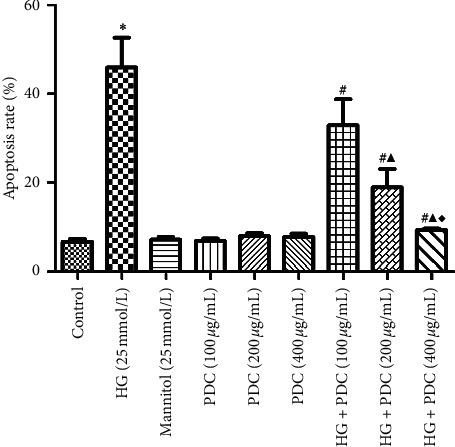
Effects of high glucose and PDC on apoptosis of HSF cells. HSF cells were incubated at high glucose concentration (25 mmol/L) or PDC (100, 200, and 400 *μ*g/mL) for 48 h. Apoptosis was detected by flow cytometry. All the data were expressed as $\bar{x}\pm S$ (*n* = 3). ^*∗*^*P* < 0.05, compared with the control group; ^#^*P* < 0.05, compared with the high-glucose group; ^▲^*P* < 0.05, compared with the high-glucose + *Dendrobium* polysaccharide (100 *μ*g/mL) group; ^♦^*P* < 0.05, compared with the high-glucose + *Dendrobium* polysaccharide (200 *μ*g/mL) group.

The results of the analysis are shown in Figure 2 and Table S2. It was determined that the apoptosis rate of the HSF cells in the high-glucose group significantly increased compared with the control group (*P*<0.05). Conversely, the apoptosis rate of the same mannitol content group was not significantly different from the control group (*P*>0.05), indicating that high glucose concentration leads to increased apoptosis of the HSF cells. Furthermore, the induced cell damage is not related to the osmotic pressure. Apoptosis rates of the PDC (100, 200, and 400 *μ*g/mL) groups were not significantly different from those of the control group (*P*>0.05). Notably, compared with the high-glucose group, the high-glucose + PDC (100, 200, and 400 *μ*g/mL) group had reduced high glucose-induced apoptosis in a concentration-dependent manner (*P*<0.05).

### 3.3. Effects of High Glucose Concentration and PDC on HSF Cell Collagen Content in the Cell Culture Fluid

**Figure 3:**
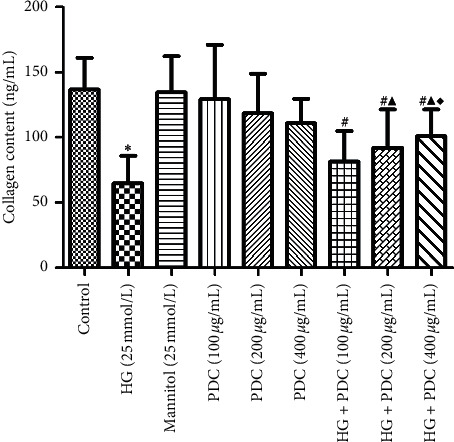
Effect of high glucose concentration and PDC on the collagen content in the cell culture fluid. HSF cells were incubated at high glucose concentration (25 mmol/L) or PDC (100, 200, and 400 *μ*g/mL) for 48 h. The cell culture fluid was collected, and the level of collagen in the culture fluid was detected by a kit method. All the data were expressed as $\bar{x}\pm S$ (*n* = 3). ^*∗*^*P* < 0.05, compared with the control group; ^#^*P* < 0.05, compared with the high-glucose group; ^▲^*P* < 0.05, compared with the high-glucose + *Dendrobium* polysaccharide (100 *μ*g/mL) group; ^♦^*P* < 0.05, compared with the high-glucose + *Dendrobium* polysaccharide (200 *μ*g/mL) group.

Compared with the control group, the collagen level in the HSF cell culture fluid of the high-glucose group significantly decreased (*P*<0.05) (Figure 3 and Table S3). Collagen levels in the cell culture fluid of the PDC (100, 200, and 400 *μ*g/mL) groups did not change compared with the control group (*P* > 0.05). Moreover, in comparison with the high-glucose group, the collagen level in the culture fluid in the high-glucose + PDC (100, 200, and 400 *μ*g/mL) groups significantly increased in a concentration-dependent manner (*P*<0.05).

### 3.4. Effect of High Glucose Concentration and PDC on the mRNA Expression of MMP-2 and TIMP-2 in the HSF Cells

**Figure 4:**
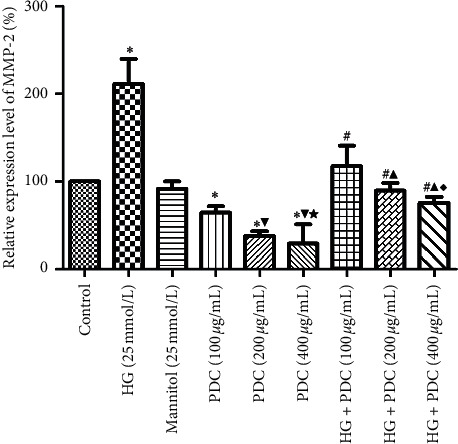
Effects of high glucose concentration and PDC on the mRNA expression of MMP-2 in HSF cells. HSF cells were incubated at high glucose (25 mmol/L) or PDC (100, 200, and 400 *μ*g/mL) for 48 h. The total RNA of the cells was collected, and the mRNA expression of MMP-2 in the cells was detected by real-time quantitative PCR. All the data were expressed as $\bar{x}\pm S$ (*n* = 3). ^*∗*^*P* < 0.05, compared with the control group; ^▼^*P* < 0.05, compared with the *Dendrobium* polysaccharide (100 *μ*g/mL) group; ^★^*P* < 0.05, compared with the *Dendrobium* polysaccharide (200 *μ*g/mL) group; ^#^*P* < 0.05, compared with the high-glucose group; ^▲^*P* < 0.05, compared with the high-glucose + *Dendrobium* polysaccharide (100 *μ*g/mL) group; ^♦^*P* < 0.05, compared with the high-glucose + *Dendrobium* polysaccharide (200 *μ*g/mL) group.

**Figure 5:**
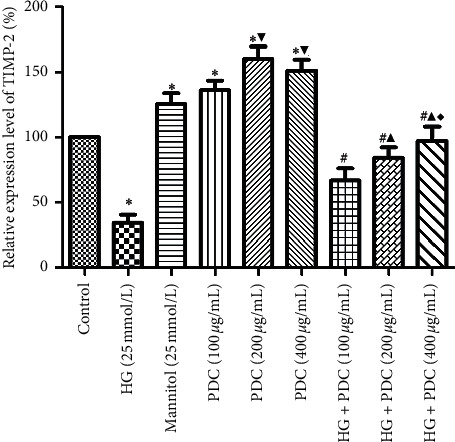
Effects of high glucose concentration and PDC on the mRNA expression of TIMP-2 in the HSF cells. HSF cells were incubated at high glucose concentration (25 mmol/L) or PDC (100, 200, and 400 *μ*g/mL) for 48 h. The total RNA of the cells was collected, and the mRNA expression of TIMP-2 in the cells was detected by real-time quantitative PCR. All the data were expressed as $\bar{x}\pm S$ (*n* = 3). ^*∗*^*P* < 0.05, compared with the control group; ^▼^*P* < 0.05, compared with the *Dendrobium* polysaccharide (100 *μ*g/mL) group; ^#^*P* < 0.05, compared with the high-glucose group; ^▲^*P* < 0.05, compared with the high-glucose + *Dendrobium* polysaccharide (100 *μ*g/mL) group; ^♦^*P* < 0.05, compared with the high-glucose + *Dendrobium* polysaccharide (200 *μ*g/mL) group.

Compared with the control group, the expression of MMP-2 mRNA significantly increased in the high-glucose HSF cells (*P*<0.05), while the expression of TIMP-2 mRNA decreased (Figures 4 and 5 and Tables S4 and S5). Compared with the control group, the PDC (100, 200, and 400 *μ*g/mL) downregulated the expression of MMP-2 mRNA and upregulated the expression of TIMP-2 mRNA in the HSF cells in a concentration-dependent manner (*P*<0.05). Compared with the high-glucose group, the expression of MMP-2 mRNA in the HSF cells in the high-glucose + PDC (100, 200, and 400 *μ*g/mL) group was notably reduced, whereas the expression of TIMP-2 mRNA increased in a concentration-dependent manner (*P*<0.05).

## 4. Discussion

The high glucose environment mimicing diabetic state can not only reduce the ability to migrate and proliferate of fibroblasts, but also increase the cell apoptosis. As previously mentioned, fibroblasts constitute the principal repair cells in wound healing and are some of the main components of the granulation tissue. They synthesize and secrete the extracellular matrix, including collagen, fibronectin, and hyaluronic acid [20–23]. Thus, in the pathological state of diabetes, increased fibroblast apoptosis inevitably hinders the healing of skin ulcers. The dynamic balance of the matrix metalloproteinase (MMP) family is abnormal during diabetes. The increase in the expression of MMPs and enhanced enzyme activity can lead to excessive degradation of the extracellular matrix, resulting in the formation of the chronic refractory wound surface. Studies have shown that MMP-2 is increased in undamaged dermal fibroblasts [24–26].

It has been demonstrated previously that, in traditional Chinese medicine, *Dendrobium officinale* could strengthen the spleen as well as nourishes the stomach, lungs, and kidneys. And it is typically used to treat chronic gastritis, hypertension, diabetes, and chronic nephritis, as well as nephropathy, and is often employed as an antitumor and antiaging agent [27, 28]. Similar substances were shown to have effects on keratinocytes or endothelial cells, which were also involved in diabetic wound healing. Mo et al. explored the effect of erianin, a bibenzyl compound extracted from *Dendrobium chrysotoxum*, on proliferation and apoptosis in HaCaT cells and demonstrated that erianin could be recognized as a potential antipsoriasis drug that inhibited proliferation and induced apoptosis of HaCaT cells through ROS-mediated JNK/c-Jun and AKT/mTOR signaling pathways [29]. Erianin was also found to induce a JNK/SAPK-dependent metabolic inhibition in human umbilical vein endothelial cells [30]. The ethanol extract of *Dendrobium chrysotoxum* Lindl ameliorates retinal angiogenesis during the development of diabetic retinopathy via inhibiting the expression of VEGF/VEGFR2 and other proangiogenic factors such as MMP-2/9 [31]. The present study found that, under high-glucose conditions, the HSF cell viability was significantly reduced, apoptosis rates considerably increased, and collagen levels were notably reduced. Furthermore, the mRNA expression of TIMP-2 decreased, while the mRNA expression of MMP-2 increased in the HSF cells. The addition of PDC (100, 200, and 400 *μ*g/mL) in HSF cells did not have a significant effect on cell viability, apoptosis, or collagen levels. However, PDC (100, 200, and 400 *μ*g/mL) could reverse the changes in the collagen level, viability, and apoptosis rate of the HSF cells caused by high glucose, with the expression of protein and TIMP-2 mRNA increased and the level of MMP-2 mRNA decreased in a concentration-dependent manner (*P* < 0.05). Hence, the present study confirms that PDC can exhibit certain protective effects on the human skin fibroblast function under high-glucose conditions. Besides, we inferred that PDC could increase the collagen synthesis of the skin fibroblasts by upregulating TIMP-2 and inhibiting the mRNA expression of MMP-2, which may be conducive to the repair of diabetic skin lesions. Further research is needed to determine the signaling pathways involved in the regulation of collagen synthesis by TIMP-2 in skin fibroblasts. The overexpression of MMP-2 in the involved psoriatic epidermis was found to be accompanied by basement membrane alterations with degraded collagen type IV [32]. Previous studies have also shown that whole-brain irradiation mediates degradation of collagen type IV by altering the balance of MMP-2 and TIMP-2 levels in the brain [33], MMPs and TIMPs are responsible for remodeling in the healthy extracellular matrix, where they are produced in a coordinated manner, and Kozaci et al. found that pro-MMP-2 levels negatively correlated with the collagen content in herniated disc material [34].

The molecular mechanisms in the pathways regulating TIMP-2 and MMP-2 expressions have been introduced in many diseases, especially diabetes, or different diseases other than diabetes. The study conducted by Ho et al. [35] found that oxidative stress induced by high glucose might be involved in the opposite effects on MMP-2 activation and TIMP-2 downregulation. This reactive oxygen species- (ROS-) dependent MMP-2 activation, in turn, mediated high-glucose-induced cell apoptosis in human umbilical vein endothelial cells (HUVECs). Besides, the transforming growth factor (TGF)/SMAD family member 3 (Smad3) pathway was found to regulate MMP/TIMP activity, inducing activation of the fibrosis mediators and suppressing the degradation of the extracellular matrix [36–38]. TIMPs could control the MMP activity, and MMP-2 could digest fibrillar collagen peptides and newly formed collagen fibers to degrade collagen [39, 40]. Furthermore, more evidence showed that TIMP-2 and MMP-2 expressions could play an important role in the development of other diseases. MMP-2, TIMP-2, and MMP-2/TIMP-2 ratios may act as biomarkers for susceptibility to systemic lupus erythematosus (SLE) [41]. Pathogenesis of chronic rhinosinusitis might also be related to the regulation of MMP-2 and TIMP-2 expressions. Steroids could inhibit smoke-regulated MMP-2 and TIMP-2 production and activation through the reactive oxygen species (ROS)/PI3K, Akt, and NF-*κ*B signaling pathways in nasal fibroblasts [42]. However, the causal relationship between MMP-2/TIMP-2 activity and collagen in skin fibroblasts remains unclear and needs to be further investigated. Selective MMP-2 inhibitors and MMP-2 knockout mice could be employed as in-depth pharmacological and genetic approaches to elucidate a mechanistic link among the MMP-2 expression, TIMP-2 expression, and changes in the collagen content in the skin fibroblasts exposed to elevated glucose.

In conclusion, our study revealed that PDC can exhibit protective effects on HSF under high-glucose conditions, which may be related to the upregulation of the TIMP-2 expression and inhibition of the MMP-2 expression, which provided new concepts for the prevention and treatment of diabetic skin ulcers or wounds using traditional Chinese medicine, including *Dendrobium officinale*.

## Figures and Tables

**Table 1:** Specific primer sequences for RT-qPCR.

Gene		Sequence (5′-3′)	Product length (bp)
TIMP-2	Forward	CCTCTGGCATCTCTTGT	423
	Reverse	ACCCCACAGCCAGCACTAT	

Bcl-2	Forward	TTCTGGGCAACA AGTATGA	436
	Reverse	GTCACTGTCCGCCAAATA	

GAPDH	Forward	TCGGACGCCTGGTTAC	199
	Reverse	CGCT CCTGGA AGATGG	

Primer design: the target gene sequence was searched in the NCBI. Primer 5 software was used to design the primer.

## Data Availability

The data generated or analyzed during this study are included in this published article. The datasets generated and/or analyzed during the current study are available from the corresponding author upon reasonable request.

## Supplementary Materials

Supplementary Materials Table S1: effects of high glucose concentration and PDC on the viability of the HSF cells. Table S2: effects of high glucose and PDC on apoptosis of HSF cells. Table S3: effect of high glucose concentration and PDC on the collagen content in the cell culture fluid. Table S4: effects of high glucose concentration and PDC on the mRNA expression of MMP-2 in HSF cells. Table S5: effects of high glucose concentration and PDC on the mRNA expression of TIMP-2 in the HSF cells.
