# Supplementary material for: Effects of Dendrobium Polysaccharides on the Functions of Human Skin Fibroblasts and Expression of Matrix Metalloproteinase-2 under High-Glucose Conditions
Source: Int J Endocrinol. 2021 Mar 8;2021:1092975. doi: 10.1155/2021/1092975 (PMC7969111; doi:10.1155/2021/1092975)
Supplement: Supplementary Materials — Table S1: effects of high glucose concentration and PDC on the viability of the HSF cells. Table S2: effects of high glucose and PDC on apoptosis of HSF cells. Table S3: effect of high glucose concentration and PDC on the collagen content in the cell culture fluid. Table S4: effects of high glucose concentration and PDC on the mRNA expression of MMP-2 in HSF cells. Table S5: effects of high glucose concentration and PDC on the mRNA expression of TIMP-2 in the HSF cells. [file 1092975.f1.docx]

**Table S1 Effects of high glucose concentration and PDC on the viability of the HSF cells**

| Group | OD value |
| --- | --- |
| Control | 0.96 ±0.07 |
| HG（25mmol/L） | 0.42±0.05^*^ |
| Mannitol（25mmol/L） | 1.05± 0.11 |
| PDC（100 μg/mL） | 0.91± 0.09 |
| PDC（200 μg/mL） | 1.05± 0.10 |
| PDC（400 μg/mL） | 0.97± 0.07 |
| HG+PDC（100 μg/mL） | 0.60±0.08^#^ |
| HG+ PDC（200 μg/mL） | 0.80±0.10^#^ ^▲^ |
| HG+ PDC（400 μg/mL） | 0.94±0.09^# ▲^^◆^ |

HSF cells were incubated at high glucose concentration (25 mmol/L) or PDC (100, 200, and 400 μg/mL) for 48 h. The MTT assay was used to detect the cell viability. All data are expressed as ‾x±S (n = 3). * P <0.05, compared with the control group; #P <0.05, compared with the high glucose group; ^▲^P <0.05, compared with the high glucose + Dendrobium polysaccharide (100 µg/mL) group; ^◆^P <0.05, compared with the high glucose + Dendrobium polysaccharide (200 µg/mL) group. All the results were verified by a Tukey HSD post hoc test.

**Table S2 Effects of high glucose and PDC on apoptosis of HSF cells**

| Group | Apoptotic rate (%) |
| --- | --- |
| Control | 6.70±0.63 |
| HG (25mmol/L) | 45.99 ± 6.73^*^ |
| Mannitol (25mmol/L) | 7.19 ± 0.59 |
| PDC (100 μg/mL) | 6.89 ± 0.53 |
| PDC (200 μg/mL) | 7.99± 0.65 |
| PDC (400 μg/mL) | 7.73 ± 0.80 |
| HG+PDC (100 μg/mL) | 32.98 ± 5.80^#^ |
| HG+ PDC (200 μg/mL) | 19.04 ± 4.01^# ▲^ |
| HG+ PDC (400 μg/mL) | 9.32 ±0.34^# ▲◆^ |

HSF cells were incubated at high glucose concentration (25 mmol/L) or PDC (100, 200, and 400 μg/mL) for 48 h. Apoptosis was detected by flow cytometry. All data are expressed as‾x±S (n = 3). *P<0.05, compared with the control group; ^#^P<0.05, compared with the high glucose group; ^▲^P<0.05, compared with the high-glucose + Dendrobium polysaccharide (100 μg/mL) group;^◆^P<0.05, compared with the high glucose + Dendrobium polysaccharide (200 μg/mL) group. All the results were verified by a Tukey HSD post hoc test.

**Table S3 Effect of high glucose concentration and PDC on collagen content in the cell culture fluid**

| Group | Collagen content (ng/mL) |
| --- | --- |
| Control | 136.77 ±24.45 |
| HG (25mmol/L) | 64.81± 21.23^*^ |
| Mannitol (25mmol/L) | 134.55 ± 27.87^#.^^▼^ |
| PDC (100 μg/mL) | 129.22 ± 42.07^#,▼^ |
| PDC (200 μg/mL) | 118.45 ± 30.43^#,▼^ |
| PDC (400 μg/mL) | 110.79 ±18.82^#,▼^ |
| HG+PDC (100 μg/mL) | 81.65± 23.09^,# ,▲^ |
| HG+ PDC (200 μg/mL) | 92.01 ± 29.43^*,#,▲,^ |
| HG+ PDC (400 μg/mL) | 100.80 ± 20.61^*,#,▲,◆^ |

HSF cells were incubated at high glucose concentration (25 mmol/L) or PDC (100, 200, and 400 μg/mL) for 48 h. The cell culture fluid was collected, and the level of collagen in the culture fluid was detected by a kit method. All data are expressed as‾x±S(n = 3). ^*^P <0.05, compared with the control group; ^▼^ P>0.05, compared with the control group; ^#^P <0.05, compared with the high glucose group; ^▲^P <0.05, compared with the high glucose + Dendrobium polysaccharide (100 μg/mL) group; ;^◆^P<0.05, compared with the high glucose + Dendrobium polysaccharide (200 μg/mL) group. All the results were verified by a Tukey HSD post hoc test.

**Table S4 Effects of high glucose concentration and PDC on mRNA expression of MMP-2 in HSF cells**

| Group | Relative expression level of MMP-2（%） |
| --- | --- |
| Control | 100.00 ± 0.00 |
| HG (25mmol/L) | 211.33 ± 28.90^*^ |
| Mannitol (25mmol/L) | 91.56 ± 8.44^*^ |
| PDC (100 μg/mL) | 64.33 ± 7.23^*^ |
| PDC (200 μg/mL) | 37.26 ± 5.52^*,^^▼^ |
| PDC (400 μg/mL) | 28.95± 21.87^*,▼,★^ |
| HG+PDC (100 μg/mL) | 117.70 ± 23.30^*,# ,▲^ |
| HG+ PDC (200 μg/mL) | 89.65 ± 8.73^*,#,▲,^ |
| HG+ PDC (400 μg/mL) | 75.40± 7.09^*,#,▲,◆^ |

HSF cells were incubated at high glucose (25 mmol/L) or PDC (100, 200, and 400 μg/mL) for 48 h. The total RNA of the cells was collected and the mRNA expression of MMP-2 in the cells was detected by real-time quantitative PCR. All data are expressed as‾x±S (n = 3). ^*^P <0.05, compared with the control group;▼P <0.05, compared with the Dendrobium polysaccharide (100 μg/mL) group; ^★^P <0.05, compared with the Dendrobium polysaccharide (200 μg/mL) group; ^#^P <0.05, compared with high glucose group; on ^▲^P <0.05, compared with the high glucose + Dendrobium polysaccharide (100 μg/mL) group;^◆^P <0.05, compared with high glucose + Dendrobium polysaccharide (200 μg/mL) group. All the results were verified by a Tukey HSD post hoc test.

**Table S5** **Effects of high glucose concentration and PDC on mRNA expression of TIMP-2 in the HSF cells**

| Group | Relative expression level of TIMP-2 (%) |
| --- | --- |
| Control | 100.00 ± 0.00 |
| HG (25mmol/L) | 34.25± 6.10^*^ |
| Mannitol (25mmol/L) | 125.57 ± 8.33^*^ |
| PDC(100 μg/mL) | 136.33 ± 7.23^*^ |
| PDC (200 μg/mL) | 160.26 ± 9.52^*,▼^ |
| PDC (400 μg/mL) | 151.29 ± 8.40^*,▼^ |
| HG+PDC (100 μg/mL) | 66.79 ± 9.33^,# ,▲^ |
| HG+ PDC (200 μg/mL) | 84.31 ± 7.90^*,#,▲,^ |
| HG+ PDC (400 μg/mL) | 97.09 ± 11.21^*,#,▲,◆^ |

HSF cells were incubated at high glucose concentration (25 mmol/L) or PDC (100, 200, and 400 μg/mL) for 48 h. The total RNA of the cells was collected, and the mRNA expression of TIMP-2 in the cells was detected by real-time quantitative PCR. All data are expressed as‾x±S (n = 3). ^*^P <0.05, compared with the control group; ^▼^P <0.05, compared with the Dendrobium polysaccharide (100 μg/mL) group; ^#^P <0.05, compared with the high glucose group; on <0.05, compared with the high glucose + Dendrobium polysaccharide (100 μg/mL) group; ^◆^P <0.05, compared with the high glucose + Dendrobium polysaccharide (200 μg/mL) group. All the results were verified by a Tukey HSD post hoc test.
